# Food-First Approach to Enhance the Regulation of Post-exercise Skeletal Muscle Protein Synthesis and Remodeling

**DOI:** 10.1007/s40279-018-1009-y

**Published:** 2019-01-22

**Authors:** Nicholas A. Burd, Joseph W. Beals, Isabel G. Martinez, Amadeo F. Salvador, Sarah K. Skinner

**Affiliations:** 1 Department of Kinesiology and Community Health, University of Illinois at Urbana-Champaign, 906 S. Goodwin Avenue, Urbana, IL 61801 USA; 2 Division of Nutritional Sciences, University of Illinois at Urbana-Champaign, Urbana, IL USA

## Abstract

Protein recommendations are provided on a daily basis as defined by the recommended dietary allowance (RDA) at 0.80 g protein/kg/day. However, meal-based, as opposed to daily, dietary protein recommendations are likely more informative given the role of the daily protein distribution pattern in modulating the post-exercise muscle protein synthetic response. Current protein meal recommendations to plateau post-exercise muscle protein synthesis rates are based on the ingestion of isolated protein sources, and not protein-rich whole foods. It is generally more common to eat whole food sources of dietary protein within a normal eating pattern to meet dietary protein requirements. Yet, there is a need to define how dietary protein action on muscle protein synthesis rates can be modulated by other nutrients within a food matrix to achieve protein requirements for optimal muscle adaptations. Recent developments suggest that the identification of an “optimal” protein source should likely consider the characteristics of the protein and the food matrix in which it is consumed. This review aims to discuss recent concepts related to protein quality, and the potential interactive effects of the food matrix, to achieve optimal protein requirements and elicit a robust postprandial muscle protein synthetic response with an emphasis on the post-exercise recovery window.

## Key Points

**Table:** 

Whole protein foods are often more than their constituent amino acids, containing other non-protein nutritive components to facilitate nutrient–nutrient interactions, modulate nutrient behavior, and/or act directly as anabolic signaling molecules.
A food-first approach to post-exercise protein intake will be beneficial for both the skeletal muscle adaptive response and diet quality for most people.

## Introduction

The ingestion of protein immediately after exercise [1] and throughout a prolonged recovery period [2, 3] is essential to stimulate muscle protein synthesis rates to facilitate remodeling and repair. Muscle protein remodeling, or the dynamic process of synthesis and breakdown, is required to remove and replace damaged proteins with new muscle proteins (reviewed in [4]). It is these exercise-induced increases in protein remodeling that provide the basis for training adaptations that lead to improved physical performance [5, 6]. While both protein synthesis and breakdown are relevant for muscle mass remodeling, exercise and feeding-induced protein synthesis provide the greatest contribution to the net anabolic response at the muscle level in healthy adults [7–9]. This notion may become more blurred at the whole body level [10]. As such, there has been much interest in the role of protein nutrition in maximizing acute changes in post-exercise muscle protein synthesis rates, and its implication on the long-term muscle adaptive response to exercise training. It is important to note that these exercise-induced increases in muscle protein synthesis rates can facilitate muscle adaptations that are either hypertrophic or non-hypertrophic in nature [4].

An area within performance nutrition that has received considerable attention is defining the optimal level of protein intake in a meal to maximally stimulate the post-exercise muscle protein synthetic response. It has been shown that the ingested protein-dose response curves of post-exercise muscle protein synthesis rates reach their breaking points, or plateau, at ~ 0.25 g protein/kg per meal in healthy young men [1, 11, 12]. In some cases, ingestion of larger protein amounts were required to induce a plateau on the dose–response curve of muscle protein synthesis rates in young adults [13]. This result was believed to relate to the amount of exercised muscle mass (i.e., full body vs. lower body resistance exercise regimes). Thus, exercise mode, intensity, and duration may differentially impact the post-exercise protein meal requirements to optimize the stimulation of muscle protein synthesis rates [13, 14]. It is evident that protein requirements are elevated above the protein recommended dietary allowance (RDA; set at 0.8 g protein/kg/day) when the goal is to optimize post-exercise muscle protein synthesis rates and remodeling [15]. However, the latter point is not surprising as the RDA values are established to prevent deficiencies and, specifically, the protein RDA only represents the minimal daily amount of protein required to consume to prevent net nitrogen (protein) loss in inactive individuals. The protein RDA, therefore, is not set as an “optimal” dietary target to maximize muscle mass. However, it serves as a starting point and helps in the establishment of a more optimal dietary allowance for protein with exercise training.

Currently there are limited data available with regards to the impact of whole food ingestion to contribute protein meal requirements to stimulate post-exercise muscle protein synthesis rates when compared to isolated protein sources. This is relevant as dietary protein is more commonly acquired through whole foods rather than ingesting isolated protein sources during the majority of meal-times. Besides supplying dietary amino acids, protein-dense whole foods often provide other important non-protein components (e.g., lipids, carbohydrates, micronutrients, and other bioactive constituents) within their food matrix that may interact and subsequently contribute to the regulation of muscle protein synthesis rates, and at the same time improve overall diet quality. In this review, we discuss the current understanding and recent advancements of protein quality and the potential contributions of the food matrix to the anabolic milieu and synergistic stimulation of muscle protein synthesis rates and remodeling with an emphasis on the post-exercise recovery period.

## Protein Quality

It is recommended to meet dietary protein intakes by ingesting highly-digestible, high-quality proteins. There are various methods available for protein quality evaluation such as the protein digestibility-corrected amino acid score (PDCAAS) and the digestible indispensable amino acid score (DIAAS) to reference proteins according to their ability to deliver target intakes of indispensable amino acids. For the past two decades, PDCAAS has been used to estimate food protein quality [16]. However, the Food and Agriculture Organization (FAO) currently recommends the DIAAS procedure [16], which takes into account that the digestibility of amino acids should be directly determined at the end of the small intestine (true ileal digestibility) and is commonly performed in growing pigs. DIAAS is an improvement upon PDCAAs that estimates protein quality based on total tract fecal digestibility with the use of rats as its model [17]. Table 1 lists the DIAAS and PDCAAS of several isolated proteins and protein dense foods. Based on the listed protein quality scores, it is evident that DIAAS improves upon PDCAAS by not truncating scores at 1.0 as well as circumventing other flaws [17–19]. As such, DIAAS should allow for an improved ranking system to avoid underestimating the anabolic potential of high(er)-quality proteins.

**Table 1:** Digestible indispensable amino acid score **(**DIAAS) and protein digestibility-corrected amino acid score (PDCASS) for isolated proteins and whole foods

	DIAAS^a^	PDCAAS (nontruncated)^b^	References^c^
Animal-derived foods			
Whey protein isolate^e^	1.00	0.99	[17]
Whey protein concentrate^e^	1.07	1.00 (1.07)	[17]
Milk protein concentrate^e^	1.20	1.00 (1.21)	[17]
Skimmed milk protein^e^	1.05	1.00 (1.12)	[17]
Whole milk powder^e^	1.16	1.00 (1.16)	[66]
Casein^e, f^	1.09	1.00 (1.20)	[67, 68]
Cow milk^e^	1.16		[66]
Sheep milk^e^	1.09		[66]
Goat milk^e^	1.24		[66]
Whole egg, boiled^e^	1.13	1.00 (1.05)	[66]
Beef^e^	1.12	1.00 (1.14)	[69]
Pork^e^	1.14	1.00	[66]
Chicken breast^e^	1.08	1.00 (1.01)	[66]
Tilapia (fish)^d^	1.00		[69]
Non-animal-derived foods			
Soya protein isolate^e^	0.84	0.93	[17]
Soya flour^e^	0.89	0.98	[17]
Wheat^e^	0.45	0.50	[17]
Pea protein concentrate^e^	0.62	0.75	[17]
Cooked peas^f^	0.58	0.60	[70]
Oat protein concentrate^e^	0.67	0.69	[71]
Cooked rolled oats^f^	0.54	0.67	[70]
Rice protein concentrate^f^	0.37	0.42	[70]
Cooked rice^f^	0.60	0.62	[70]
Rye^e^	0.48	0.59	[69]
Barley^e^	0.47	0.59	[69]
Peas^e^	0.65	0.79	[69]
Sorghum^e,f^	0.29	0.29	[72, 73]
Cooked kidney beans^f^	0.59	0.65	[70]
Roasted peanuts^f^	0.43	0.51	[70]
Corn based breakfast cereal^f^	0.01	0.08	[70]

Only values that used the scoring patterns for children older than 3 years, adolescents, and adults were selected

^a^Values for DIAAS were calculated from the ileal digestibility of amino acids

^b^Values for PDCAAS were calculated from the total tract digestibility of crude protein

^c^All values for DIAAS and PDCAAS were selected in humans, if available, growing pigs, or in growing rats in that order

^d^Measured in humans

^e^Measured in pigs

^f^Measured in rats

While DIAAS provides a better method to define protein quality in terms of the relative digestible content of the IAAs and the amino acid requirement, much of the research into DIAAS is limited to isolated protein sources and/or raw feedstuffs for livestock production. This is noteworthy as cooking (heat treatment) of protein foods can modify digestive kinetics and metabolism of dietary proteins [20, 21]. In addition, DIAAS does not attempt to consider how the scores translate into optimizing more downstream physiological targets of interest to a physically active person or athlete. The primary metabolic action of dietary protein-derived amino acids is to stimulate whole body and muscle protein synthesis to support a positive net protein balance [22]. As such, it is important to couple assessments of dietary protein quality with other metabolic correlates, such as protein synthesis, in order to more comprehensively characterize the anabolic potential of dietary proteins to augment the quality and quantity of muscle protein. Moreover, it has been demonstrated that exercise may directly impact protein digestibility and the subsequent release of dietary protein derived amino acids into the circulation [23]. Thus, it is likely important to consider the consequences of prior exercise on protein digestibility/quality especially for more physically active populations such as athletes involved in regular training or competition.

The invasive nature of determining DIAAS in vivo in humans precludes the ability of this method to be coupled with an exercise setting [24]. As such, identifying approaches that are more readily adaptable to an exercising human to allow for assessment of protein digestibility would be useful, especially given the interactive nature of protein nutrition and exercise on whole body and muscle protein metabolic responses. Intrinsic labeling of protein foods with stable isotope amino acids has been useful to provide an index of food protein digestibility against the background of exercise in vivo in humans [25–27]. This method combines primed constant infusion methods with specifically produced labeled food proteins to assess the amount and speed of release of dietary protein derived amino acids in the circulation. Table 2 lists the different protein foods that have been intrinsically labeled to determine the amount of dietary protein-derived amino acids (usually leucine or phenylalanine) that appeared in the circulation, expressed as a percentage, after their ingestion. It is important to recognize that the amino acid labeled in the ingested food may have a direct impact on the amino acid availability expressed in Table 2. For example, it has been shown that there are differences in postprandial splanchnic handling of leucine versus phenylalanine [28], which impacts their appearance rates and concentrations in the blood. In general, there is greater selective splanchnic uptake of phenylalanine when compared to leucine. Overall, plasma dietary amino acid availability measurements can be coupled with measurements of muscle protein synthesis rates to provide additional insight into the anabolic potential of the ingested food (Table 2).

**Table 2:** Protein-derived amino acid availability in the circulation and postprandial rates of muscle protein synthesis (MPS) after ingestion of isolated protein sources and whole foods healthy young and older adults

	Protein amount (g)	Dietary amino acid availability^a^	MPS response^b^	References
Intrinsically labeled food studies				
Casein	20	55% (Phe)	1.51^d^	[74]
Beef^c^	30	64% (Phe)	1.90^d^	[33]
Egg white^c^	18	66% (Leu)	1.90^e^	[35]
Whey	35	59% (Phe)	2.09^d^	[75]
Skim milk^c^	30	57% (Phe)	2.37^d^	[33]
Whole egg^c^	18	68% (Leu)	2.70^e^	[35]
Whey	20	58% (Phe)	No basal	[76]
Casein	20	53% (Phe)	No basal	[76]
Casein hydrolysate	20	55% (Phe)	No basal	[76]
Non-labeled food studies				
Wheat protein hydrolysate	60		1.40^d^	[31]
Soy^c^	40		1.40^d^	[77]
Pork	36		1.63^d^	[34]
Beef^c^	36		2.00^d^	[78]
Milk protein concentrate	20		2.48^d^	[79]
Whey	20		3.00^d^	[79]

^a^Fractions of dietary protein derived amino acids that appeared in the circulation (percentage) throughout 0–5 h or 0–6 h postprandial periods. The data were based on an orally ingested leucine (Leu) or phenylalanine (Phe) tracers intrinsically labeled into food sources and designated by their respective amino acid in parentheses

^b^Postprandial rates of MPS expressed as fold change from reported basal rates (when available)

^c^Indicates protein ingestion after an acute bout of exercise

^d^Indicates rates of MPS were measured using labeled phenylalanine tracer incorporation in muscle tissue

^e^Indicates rates of MPS were calculated using labeled leucine tracer incorporation into muscle tissue

**Fig. 1:**
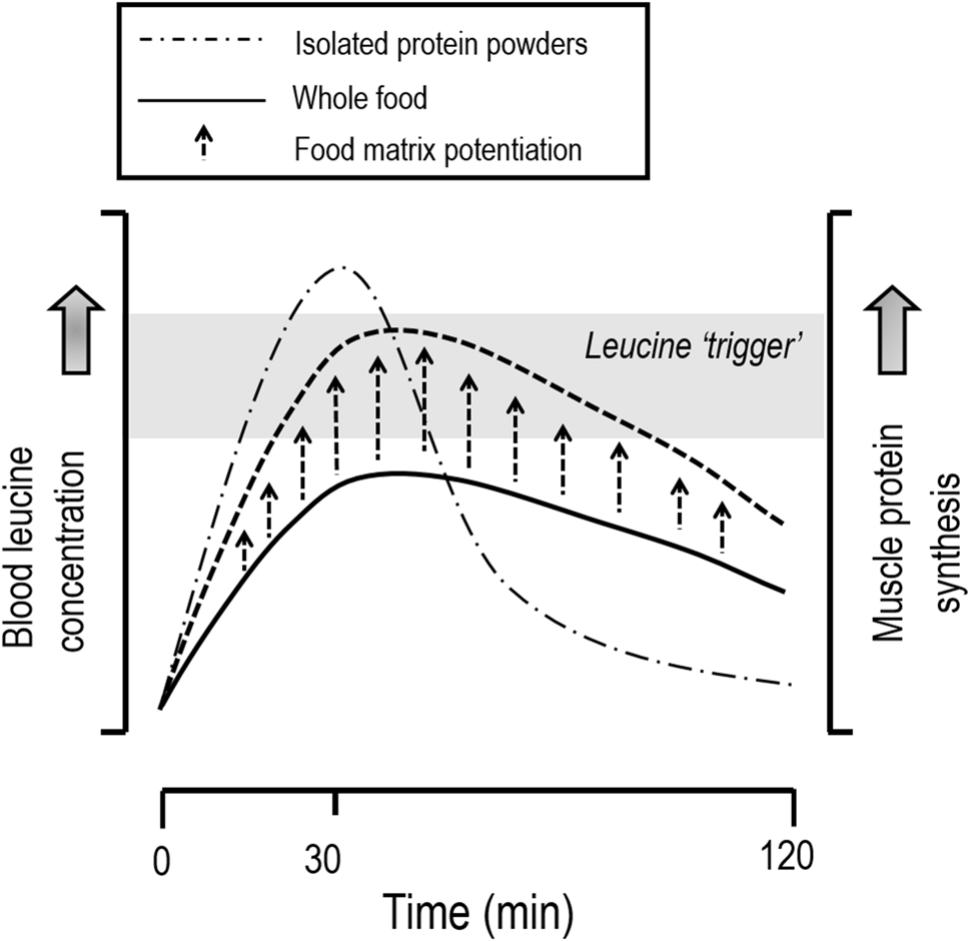
The “leucine trigger” hypothesis. The ingestion of an isolated protein source (e.g., whey) results in a rapid rise in plasma leucine concentrations, which is superior in terms of amplitude when compared to whole food sources of protein, and corresponds to the extent of stimulation of muscle protein synthesis rates [29]. However, we hypothesize that the interaction of non-protein nutritive components with dietary amino acids (food matrix effects) has a direct effect on post-exercise muscle protein synthesis rates. Overall, the leucine trigger hypothesis is probably highly relevant when ingesting isolated protein fractions, but is less applicable towards the muscle protein synthetic response when ingesting whole food sources of protein, especially in healthy adults [33, 35]

It is clear that there is limited information on how a wide variety of protein food sources stimulate postprandial muscle protein synthesis rates. Moreover, the majority of studies that have assessed the impact of protein nutrition on the stimulation of postprandial muscle protein synthesis rates have focused on isolated protein sources [29–31]. Based on these studies of isolated protein sources, such as isolated whey, micellar casein, and soy fractions [29, 32], the leucine “trigger” hypothesis was developed (Fig. 1). This leucine trigger hypothesis suggests that a rapid rise (within ~ 60–90 min) in blood leucine concentrations in close temporal proximity to an exercise bout after protein ingestion is most anabolic for stimulating post-exercise muscle protein synthesis rates, as observed with ingestion of leucine-rich isolated protein sources (Fig. 1). However, it would seem likely that there is not an absolute blood leucine concentration that serves as a “maximum switch on” for the post-exercise muscle protein synthetic response, but rather a step-wise increase in muscle protein synthesis rates with increasing blood leucine concentrations, which would lead to an eventual plateau in muscle protein synthesis rates with higher ingested protein amounts [1]. Interestingly, however, the ingestion of whole foods is also potent for the stimulation of post-exercise muscle protein synthesis rates despite not facilitating a rapid rise in leucinemia during the immediate post-exercise period [33]. Specifically, the ingestion of protein-dense whole foods results in a prolonged release of dietary amino acids into the circulation with plasma amino acid concentration values peaking at ~ 120 min of the postprandial period in healthy adults [34]. Thus, the leucine “trigger” hypothesis may be more relevant after the ingestion of isolated protein sources as opposed to whole food sources of protein. Specifically, other non-protein components within the whole food matrix may likely influence the regulation of post-exercise muscle protein synthesis rates [33, 35]. Nonetheless, there is still much to learn with respect to how food matrices containing protein interact to affect protein quality/digestibility and its implications for the post-exercise stimulation of muscle protein synthesis rates (discussed in Sect. 3).

## Food Matrix

In general, the nutritional quality of food is primarily based on the relative quantities of each individual nutritional component (e.g., protein, carbohydrates, lipids, and micronutrients). This notion has certainly been true when assessing the role of dietary protein in post-exercise muscle remodeling processes in humans as most studies have used isolated protein fractions. It has been shown that amino acids, particularly the essential amino acids [36], have potent anabolic properties towards the stimulation of muscle protein synthesis rates in vivo in humans. However, the holistic properties of foods and their potential influence on post-exercise muscle protein remodeling and repair has not been extensively studied. The food matrix refers to the overall chemical dynamics of food, which includes how various food components are structured and interact [37]. Emerging evidence seems to suggest there are potential interactions occurring within a food matrix (i.e., food synergy [38]) that modulate various metabolic processes (including muscle protein synthesis). In other words, the ingestion of specific whole foods, and the associated nutrient–nutrient interactions, possibly facilitates a stronger anabolic effect than the individual actions from each individual food component.

Elliot et al. [39] demonstrated that whole milk ingestion (627 kcals; 8 g protein, 8 g fat, and 11 g carbohydrate) consumed 1 h after resistance exercise stimulated greater amino acid uptake across the leg when compared to fat-free milk (377 kcals; 9 g protein, 0.6 g fat, and 12 g carbohydrate) or iso-caloric amounts of fat-free milk (626 kcals; 14.5 g protein, 1 g fat, and 20 g carbohydrate) in healthy men and women. Likewise, van Loon’s research group demonstrated a differential temporal stimulation of post-exercise muscle protein synthesis rates after ingestion of skim milk (30 g protein, 31 g carbohydrate, and 0.4 g fat) versus iso-nitrogenous amounts of beef (30 g protein, 0.7 g carbohydrate, and 4.6 g fat) in young men [33]. Specifically, this work demonstrated that skim milk ingestion elicited a greater stimulation of post-exercise muscle protein synthesis rates during the early (0–2 h) recovery phase when compared to beef ingestion. The greater anabolic potential on muscles during the early recovery period after skim milk ingestion occurred despite beef ingestion inducing a more rapid protein digestion and amino acid absorption rates, which ultimately facilitated more dietary amino acids being available in the circulation in the beef condition [33]. These data are interesting as they highlight that commonly assumed anabolic characteristics of an ingested protein source, such as the higher peak amplitude of leucinemia with beef ingestion, do not universally translate into a greater early muscle protein synthetic response when compared to whole food (milk) ingestion in healthy young men.

From these studies, it is not possible to elucidate the food component(s), or mechanism, within the dairy matrix that may have contributed to the differential regulation of post-exercise muscle protein synthesis rates between the ingested food sources. Interestingly, studies have demonstrated that co-ingestion of micellar casein with individual food components such as milk fat [40], carbohydrates [41], or milk serum ([42]; mixture of 10% lactose, 0.3% protein, 0.06% fat, and 1.1% minerals) does not further augment the postprandial muscle protein synthetic response when compared to ingestion of micellar casein alone. It is worth noting, however, that these studies were conducted at rest, and perhaps an exercise stimulus may be required to create a more physiologically relevant interaction between dietary amino acids and the non-protein components of the whole food at the muscle level. Nonetheless, it would seem that superior post-exercise muscle protein synthetic responses observed with whole milk [39] or skim milk [33] ingestion were not related to these specific food components. Instead, it is possible that the specific effect of a dairy matrix on the regulation of post-exercise muscle protein synthesis rates cannot be attributed to an individual nutrient and is dependent on the sum and interaction of all its nutrients. Moreover, a dairy matrix may differ between specific dairy products (full-fat vs. low-fat products such as yogurt, cheese, etc.) and between products produced from grass-fed versus grain-fed dairy cows [43]. For example, it has been suggested ingesting milk collected from grass-fed cows may confer greater health benefits (i.e., reduced risk of cardiovascular disease) when compared to milk collected from grain-fed dairy cows likely due to the manipulation of the fatty acid composition of the dairy matrix [44]. Thus, it is likely possible to manipulate the matrix of foods either with food fortification techniques or directly by altering feeding approaches within cows to impact human health.

**Fig. 2:**
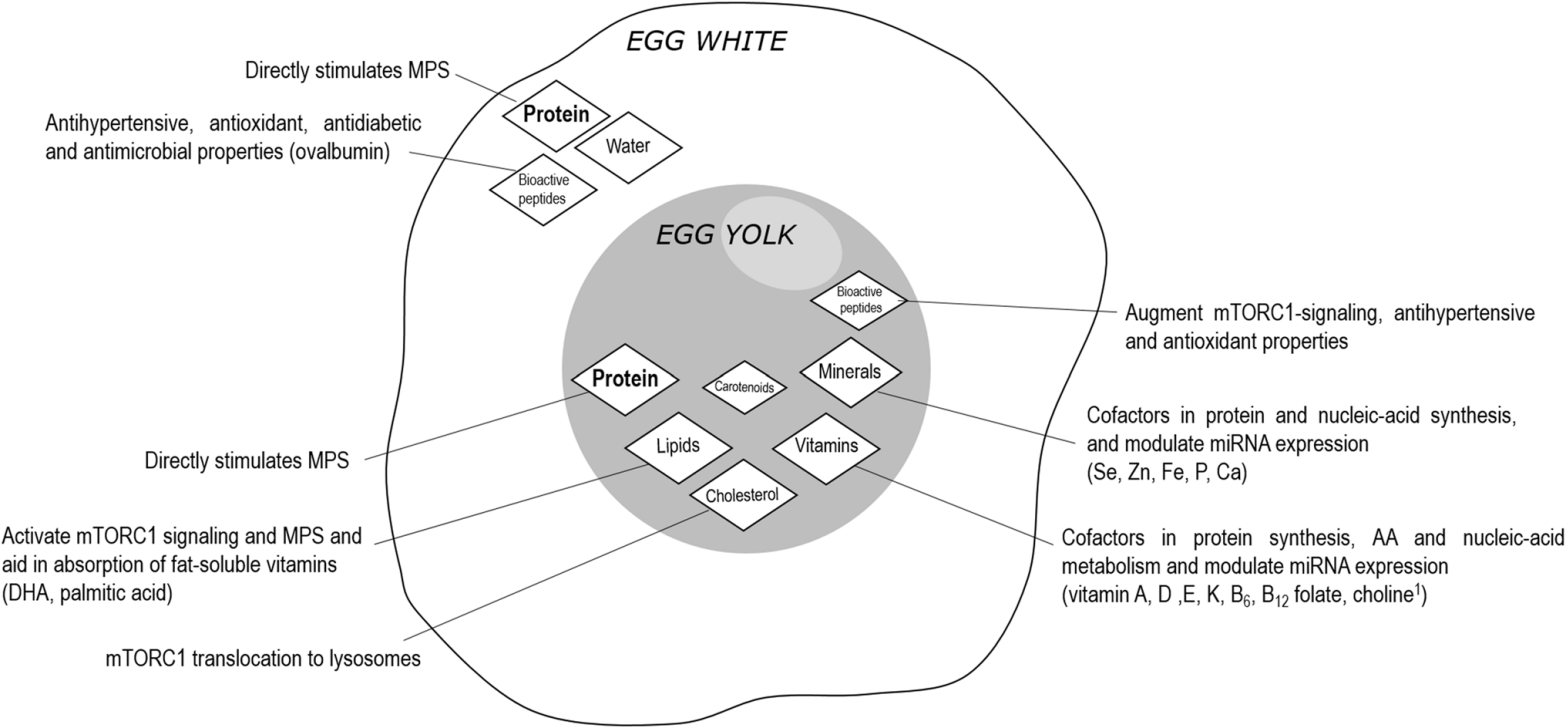
The whole egg matrix is rich in high-quality dietary protein, lipids, vitamins, and minerals when compared to the egg white matrix. While dietary amino acids are the main precursors for protein synthesis, the non-protein components of the whole egg, which are largely contained in the yolk, may have a role in various aspects of the regulation of muscle protein synthesis rates (MPS). These non-protein components include: cholesterol being involved in translocation of mTORC1 to the lysosomes [80], lipids [81], vitamins [82, 83], minerals [84], and other bioactive components [85, 86] serving to facilitate nutrient sensing mechanisms in muscle tissue. Thus, the interaction of nutrients within whole foods to support post-exercise MPS is likely greater than each respective nutrient in isolation. We propose that food matrix effects should be considered when defining optimal protein intakes to stimulate post-exercise MPS and remodeling. *mTORC1* mammalian target of rapamycin complex 1, *DHA* docosahexaenoic acid, *miRNA* micro-ribonucleic acid, *AA* amino acids. ^1^Indicates vitamin-like nutrient

Our research group has recently contributed to the concept that food matrix effects may influence the post-exercise stimulation of muscle protein synthesis rates and remodeling. Specifically, we assessed the impact of the ingestion of whole eggs or iso-nitrogenous amounts of egg whites on the stimulation of muscle protein synthesis rates during recovery from resistance exercise in healthy young men [35]. We demonstrated that the post-exercise muscle protein synthetic response was more strongly stimulated after the ingestion of whole eggs versus egg whites. Interestingly, the difference in the post-exercise stimulation of muscle protein synthesis rates between the whole egg and egg white conditions was not related to the postprandial plasma leucine availability, plasma insulin concentrations, muscle amino acid transporter content, uptake of dietary leucine into muscle, or muscle anabolic signaling pathway phosphorylation [35]. Indeed, the egg white consists of water and protein with the remainder consisting of trace amounts of carbohydrate and lipids. However, the whole egg consists of a food matrix that is rich in high quality protein, lipids, vitamins, and minerals. More work is required to confirm, but it is interesting to speculate that the whole egg matrix may be interacting to create a food synergy to support a greater post-exercise muscle protein synthetic response when compared to the egg white. For example, Fig. 2 illustrates the food components within the white and yolk portions of a whole egg and their potential contribution to the stimulation of post-exercise muscle protein synthesis rates. It is evident that proteins (amino acids) are the main precursors for muscle protein synthesis; however, other non-protein components may influence how dietary amino acids are used for protein synthesis by aiding in protein translation. Similar to dairy products, the egg matrix can also be altered through manipulation of either feed composition [45] or living conditions (cage-raised or free-range [46]) of laying hens.

Overall, the significance of food matrix manipulations and nutrient–nutrient interactions for the post-exercise stimulation of muscle protein synthesis rates is not known. However, it is important to identify more sustainable strategies for protein nutrition in modern society to compensate for the increased demand from a growing population [47] and the apparent elevated protein meal requirements to maximize muscle protein anabolism especially in people with active lifestyles [1, 13] when compared to the protein RDA. The ingestion of whole foods, with a matrix rich in dietary proteins, macro- and micro-nutrients, may be a potential dietary strategy to more efficiently utilize dietary amino acids for postprandial muscle protein accretion. However, this hypothesis still requires rigorous testing.

## Exercise, Gut Permeability, and Implications of the Food Matrix

Exercise has the potential to directly impact gastrointestinal (GI) function. This is significant as proper GI function is necessary to sustain exercise performance as well as promote substrate delivery to support glycogen re-synthesis and protein synthesis during recovery from exercise. An abundance of evidence has demonstrated that there is reduced GI barrier function (permeability) and potentially mucosal disruption as a result of acute exercise [14, 23, 48–58]. These finding of alterations in GI physiology have been demonstrated in a variety of settings including running [14, 49, 50, 52, 57, 58], cycling [48, 53–56], resistance exercise [23], and prolonged endurance exercise [51]. Moreover, there appears to be an effect of exercise intensity on the magnitude of the increased GI permeability [57]. These alterations in GI physiology appear to be related to splanchnic hypo-perfusion during the bout of exercise [56]. Also, a recent study indicated that mucosal disruption may explain some, but not all, of the exercise-induced alterations in intestinal permeability [49]. Evidence suggests, however, that food/nutrient ingestion may lead to improvements in the GI permeability response to exercise [14, 52, 54]. Specifically, increased concentrations of small intestine-derived fatty acid binding protein (I-FABP), which is often used as a bio-marker of intestinal injury, in the circulation are detectable for several hours during recovery from a 2-h bout of treadmill running [52] or after moderate intensity cycling [54]. By contrast, when food is consumed post-exercise, GI permeability returned to baseline much more rapidly [14]. In fact, when nutrition is consumed concurrently with exercise, it has been shown that gut damage markers are unaltered during or after 2 h of treadmill running as compared with consuming only water [52]. It has also been previously demonstrated that nutrient provision can ameliorate altered gut barrier function and prevent epithelial apoptosis [59]. Collectively, these data highlight that there is likely an interplay between exercise and the gut, which will likely affect the handling of nutrition during recovery from exercise.

What is noteworthy is that exercise prior to food ingestion has the potential to alter food digestion and absorption of nutrients within the food matrix. For example, it has been shown that large food-derived peptides can cross the epithelial barrier during and after exercise [53], which is uncharacteristic of normal digestion [60]. Thus, exercise has the potential to create an avenue, via increased gut permeability, for bio-active food constituents, or non-protein components, within a food matrix to enter the circulation. In addition to this, small pieces of genetic regulatory material (microRNAs and RNA) have been shown to survive digestion [61–63], which may be elevated during acute exercise recovery due to greater GI permeability. Importantly, these compounds have demonstrable effects on gene expression in host cells [62, 64]. However, there is a great deal of research still needed in this area to better define how exercise may assist in the transfer of large peptides, and/or non-nutrient food components, after food ingestion and their effects during post-exercise recovery.

## Conclusion

Dietary protein ingestion immediately after exercise [65] and throughout a prolonged recovery (≥ 1 day [2]) further increases muscle protein synthesis rates to facilitate non-hypertrophic or hypertrophic protein remodeling when compared to feeding alone. Current protein recommendations to maximize the post-exercise muscle protein synthetic response are based on isolated protein sources, but suggest that protein meal requirements are elevated when compared to the protein RDA [1, 12, 13]. Recent studies demonstrate a developing role of the food matrix in modulating the post-exercise muscle protein synthetic response [33, 35]. Specifically, it seems that the ingestion of protein-dense whole foods, and the interaction of their non-protein nutritive components, can likely potentiate the use of dietary amino acids for post-exercise muscle protein synthesis rates. However, it is unknown whether different food matrices (e.g., dairy matrix vs. egg matrix), fortification of a food matrix (e.g., manipulation of lipid, vitamin, or mineral content), or food combinations can be utilized to differentially impact the post-exercise muscle protein synthetic response and overall protein requirement. Moreover, exercise and its subsequent impact on increased GI permeability may facilitate the transfer of non-protein components and protein peptides within the food matrix to modulate the post-exercise muscle adaptive response [53]. Thus, a food-first approach to post-exercise protein intake will be beneficial for both the skeletal muscle adaptive response and diet quality for most people. Ultimately, sports dietitians will need to consider the typical eating pattern (animal- vs. plant-based diets) and travel/training schedule of an athlete when developing meal plans as this is necessary when identifying whether to incorporate whole foods, dietary supplements, or both.
